# Therapeutic effect of adalimumab in the treatment of intestinal Behçet's disease in children

**DOI:** 10.3389/fped.2025.1619065

**Published:** 2025-06-30

**Authors:** Tianjiao Gao, Xiaoxia Ren, Yanan Han, Fengfan Wang, Bianhua Liu, Ying Fang

**Affiliations:** Department of Gastroenterology, Xi’an Children’s Hospital, Xi’an, China

**Keywords:** intestinal ulcers, children, adalimumab, biologic therapy, TNF-α inhibitor, inflammatory markers, intestinal Behçet's disease

## Abstract

**Objective:**

Intestinal Behçet's disease is a rare but severe manifestation of Behçet's disease, particularly in pediatric populations. Currently, there is no cure for intestinal Behçet's disease, and the goal of treatment is to control acute episodes and reduce inflammation. Adalimumab, a tumor necrosis factor-alpha (TNF-α) inhibitor, has shown promise in adult patients, but data on its efficacy and safety in children are limited. The aim of this study was to evaluate the efficacy and safety of adalimumab in the treatment of intestinal Behçet's disease in children.

**Methods:**

A retrospective analysis was conducted on 8 children with intestinal Behçet's disease treated with adalimumab at Xi'an Children's Hospital from January 2021 to December 2024. Clinical symptoms, endoscopic findings, and inflammatory markers were assessed before and after treatment. Efficacy was evaluated on the basis of symptom resolution, ulcer healing, and changes in inflammatory markers.

**Results:**

The cohort included 4 males and 4 females, with an average age of 11.9 ± 1.4 years and a disease course of 10.5 (6.75, 22.5) months. All patients presented with gastrointestinal symptoms, including abdominal pain (*n* = 7), vomiting (*n* = 1), and complications such as intestinal obstruction (*n* = 3) and perforation (*n* = 1). Colonoscopy revealed ileocecal ulcers in 7 patients and only terminal ileal ulcers in 1 patient. After treatment with adalimumab, complete resolution of clinical symptoms was observed in all patients. Gastrointestinal endoscopy revealed that 6 patients had completely healed ulcers, while the remaining 2 patients had ulcers that were reduced to 50% of their original size. The levels of inflammatory markers significantly decreased, with the erythrocyte sedimentation rate (ESR) decreasing from 44.63 ± 43.48 mm/h to 10.50 ± 7.65 mm/h (*p* = 0.046) and the high sensitivity C-reactive protein (hs-CRP) level decreasing from 43.87 ± 39.10 mg/L to 0.96 ± 0.67 mg/L (*p* = 0.017). On the basis of clinical symptoms and endoscopic findings, 6 patients (75%) achieved complete remission, and 2 patients (25%) showed improvement. Adalimumab was well tolerated, with only one case of mild eczema reported.

**Conclusion:**

Adalimumab is safe and effective in the treatment of intestinal Behçet's disease in children.

## 1 Introduction

Behçet's disease is a chronic, relapsing systemic vasculitis characterized by recurrent oral and genital ulcers, skin lesions, and uveitis. It can also affect the nervous system, heart, blood vessels, gastrointestinal tract, lungs, kidneys, and joints. Behçet's syndrome is better characterized as a syndrome rather than a distinct disease entity, given its complex etiopathological mechanisms and variable clinical manifestations. Its heterogeneity requires a wide range of diagnostic methods and complex differential diagnostic capabilities (1). When the gastrointestinal tract is involved, it is referred to as intestinal Behçet's disease (2, 3). Intestinal Behçet's disease primarily affects young adults, accounting for 10%–50% of Behçet's disease cases, with the ileocecal region being the most commonly affected site. Endoscopic findings typically reveal deep ulcers, and the main clinical symptoms include abdominal pain and diarrhea. Severe cases may lead to intestinal perforation, bleeding, and even death, resulting in a poor prognosis (4, 5). While intestinal Behçet's disease predominantly occurs in adults, pediatric cases are increasingly recognized but remain understudied, posing diagnostic and therapeutic challenges given their atypical presentations and overlapping features with other inflammatory bowel disorders (1).

The management of Behçet's disease is complex and primarily aims to control inflammation and prevent complications (1). Corticosteroids and immunosuppressants are commonly used, but some patients have a poor response. Recent studies have shown that biologic agents are effective for refractory or recurrent cases (6). Tumor necrosis factor-alpha (TNF-α) is widely recognized as a key player in Behçet's disease. This is evidenced by increased levels of TNF-α mRNA in the serum and affected tissues of Behçet's disease patients (7). Furthermore, soluble TNF receptors 1 and 2 are generated at inflammatory sites and correlate with arthritis activity in Behçet's disease, highlighting TNF-α as a promising therapeutic target (8). Adalimumab, a TNF-α inhibitor, has shown promise in treating adult intestinal Behçet's disease in several studies (9, 10).

However, despite its established benefits in adults, data on the use of adalimumab in treating pediatric intestinal Behçet's disease are rare. Current evidence is limited to small case series or is extrapolated from adult studies. Given the aggressive disease course in pediatric patients and the potential for growth impairment due to chronic inflammation, identifying effective therapies is urgent. This single-center retrospective study evaluated the efficacy and safety of adalimumab in children with intestinal Behçet's disease. We specifically assessed (i) clinical and endoscopic response rates, including ulcer healing and symptom resolution; (ii) changes in inflammatory markers as measurable indicators of disease activity; and (iii) adverse events to establish safety profiles in this vulnerable population. By addressing these objectives, we aim to provide evidence-based guidance for the use of adalimumab in treating pediatric intestinal Behçet's disease, thus bridging a critical gap between adult and child therapeutics.

## 2 Materials and methods

### 2.1 Study population

This study was a single-center, retrospective cohort analysis conducted at Xi'an Children's Hospital from January 2021 to December 2024. The study population included children diagnosed with intestinal Behçet's disease who were treated with adalimumab. The diagnosis of intestinal Behçet's disease was based on the 2014 International Criteria for Behçet's Disease (ICBD) (11) and the 2009 Korean Behcet's Disease Collaboration Group proposed diagnostic criteria for intestinal Behçet's disease (12). The data included clinical manifestations before and after treatment, endoscopic and pathological findings, imaging studies, laboratory tests, medication, treatment response, adverse events, and outcomes.

The inclusion criteria were as follows: children aged ≤14 years with a confirmed diagnosis of intestinal Behçet's disease; children who received adalimumab treatment; and children whose complete clinical, endoscopic, and laboratory data were available before and after treatment. The exclusion criteria were as follows: severe liver or kidney dysfunction (Child‒Pugh class C score, estimated glomerular filtration rate <30 ml/min/1.73 m^2^); active tuberculosis, inflammatory bowel disease, or nonsteroidal anti-inflammatory drug (NSAID)-induced gastrointestinal ulcers; uncontrolled bacterial or viral infections; and active viral hepatitis.

This study, which followed all the requirements of the Declaration of Helsinki, was ethically approved by the review board of Xi'an Children's Hospital (approval number: 2024-019-02). Informed consent was obtained from the guardians of the children.

### 2.2 Diagnostic procedures

#### 2.2.1 Clinical evaluation

(i) Comprehensive medical history and physical examination focusing on gastrointestinal symptoms (e.g., abdominal pain, diarrhea) and extraintestinal manifestations (e.g., oral/genital ulcers, skin lesions, ocular lesions).
(ii) Pathergy tests were performed when clinically indicated.

#### 2.2.2 Endoscopic and imaging studies

(i) Colonoscopy: All patients underwent colonoscopy to assess the presence, location, and characteristics of the intestinal ulcers. Biopsies were taken from ulcer edges and surrounding mucosa for histopathological analysis.
(ii) Histopathology: Biopsy samples were evaluated for acute/chronic inflammation, ulceration, and vasculitis; other causes (e.g., Crohn's disease, tuberculosis) were excluded.
(iii) Abdominal CT/MRI: Imaging was performed to evaluate complications (e.g., obstruction, perforation) and disease extent.

### 2.3 Data collection

Data were extracted from electronic medical records and included the following:

(i) Demographics: Age, sex, disease duration, and previous treatments.
(ii) Clinical manifestations: Gastrointestinal symptoms, extraintestinal manifestations, and complications.
(iii) Endoscopic findings: Ulcer characteristics (location, size, number) and histopathology reports.
(iv) Laboratory tests: Inflammatory markers, complete blood count, and liver/kidney function tests.
(v) Imaging studies: Abdominal/pelvic CT scans were used to assess intestinal wall thickening or complications.

### 2.4 Treatment methods

Adalimumab was administered according to weight-based dosing guidelines (9):

(i) 17 kg to <40 kg: initial dose (Day 1) of 80 mg; second dose two weeks later (Day 15) of 40 mg; two weeks later (Day 29) begin a maintenance dose of 20 mg every other week.
(ii) ≥40 kg: initial dose (Day 1) of 160 mg; second dose two weeks later (Day 15) of 80 mg; two weeks later (Day 29) begin a maintenance dose of 40 mg every other week.

### 2.5 Efficacy assessment

The efficacy of adalimumab in treating intestinal Behçet's disease was evaluated on the basis of clinical symptoms and endoscopic findings (10): (i) no change/worsening: symptoms severely affect daily life, with ulcers shrinking less than 50% or enlarging; (ii) improvement: symptoms mildly affecting daily life, with ulcers shrinking to 25%–50% of their original size; (iii) significant improvement: symptoms not affecting daily life, with ulcers shrinking to less than 25% of their original size; (iv) complete remission: no clinical symptoms, with complete ulcer healing on endoscopy.

### 2.6 Statistical analysis

Statistical analysis was performed using SPSS 21.0 software. Categorical data are expressed as percentages, and continuous data are expressed as the mean ± standard deviation (SD) or median (interquartile range) depending on their distribution. The normality of the data distribution was assessed using the Kolmogorov‒Smirnov test. For normally distributed data, the *t* test was used, with *p* < 0.05 considered statistically significant.

## 3 Results

### 3.1 Baseline patient characteristics

A total of 8 children with intestinal Behçet's disease were included, comprising 4 males and 4 females, with an average age of 11.9 ± 1.4 years and a disease course of 10.5 (6.75, 22.5) months. All patients had no history of tuberculosis, inflammatory bowel disease, or other confounding conditions.

### 3.2 Clinical manifestations before Adalimumab treatment

#### 3.2.1 Gastrointestinal involvement

All 8 children exhibited gastrointestinal symptoms, with 7 patients (87.5%) presenting with abdominal pain (3 of whom had concurrent diarrhea) and 1 patient (12.5%) presenting with vomiting. Complications, including intestinal obstruction (*n* = 3) and intestinal perforation (*n* = 1), were observed in 3 patients (37.5%). Patient 2 underwent surgical resection of the affected bowel segment (including the ulcer segment) and ileostomy due to intestinal perforation complicated by obstruction. Imaging studies, including abdominal and pelvic CT scans, revealed localized intestinal wall thickening in 7 patients, which was consistent with active inflammation (Table 1).

**Table 1:** Clinical manifestations in children with Behcet's disease before adalimumab treatment.

Patient	Digestive system manifestations			Extraintestinal manifestations
	Complications	Endoscopic findings		
Patient 1	None	Ileocecal region	Single large circumferential ulcer	Oral ulcers, vulvar ulcers, perianal ulcers
Patient 2	Intestinal perforation, Intestinal obstruction	Ileocecal region	Single large circumferential ulcer	Oral ulcers, vulvar ulcers
Patient 3	None	Ileocecal region	Multiple deep, round or oval-shaped ulcers	Oral ulcers, penile ulcers
Patient 4	None	Ileocecal region	Single large circumferential ulcer	Oral ulcers, penile ulcers, pseudofolliculitis
Patient 5	Intestinal obstruction	Duodenum, ileocecal region	Large ulcer in the duodenal bulb, single large circumferential ulcer in the ileocecal region	Oral ulcers, positive pathergy test, perianal ulcers
Patient 6	None	Esophagus, ileocecal region	Two longitudinal ulcers in the esophagus, single ulcer in the ileocecal region	Oral ulcers, pseudofolliculitis, positive pathergy test
Patient 7	Intestinal obstruction	Ileocecal region	Single semi-circumferential ulcer	Oral ulcers, perianal ulcers
Patient 8	None	Terminal ileum	Single round aphthous ulcer	Oral ulcers, perianal ulcers, erythema nodosum

Endoscopic evaluation revealed ileocecal ulcers in 7 patients, with 4 patients showing large or giant ulcers. One patient had isolated terminal ileal ulcers (Table 1). A pathological examination of biopsy samples confirmed acute and chronic mucosal inflammation with ulcer formation in all patients.

#### 3.2.2 Extraintestinal manifestations

All patients presented with extraintestinal manifestations before adalimumab treatment, with oral ulcers being the most common (100%). Other manifestations included vulvar ulcers (*n* = 2), penile ulcers (*n* = 2), perianal ulcers (*n* = 4), and skin lesions (*n* = 3). A positive pathergy test was observed in 2 patients (Table 1).

### 3.3 Preadalimumab treatment

Before adalimumab treatment, 6 children were treated with prednisone (0.75–1 mg/kg·d), and 3 children were treated with immunosuppressants (thalidomide, 1.5–2.5 mg/kg·d). Despite these treatments, all patients exhibited persistent or recurrent symptoms, highlighting the refractory nature of their disease. The symptoms of all 8 children severely affected their daily life.

### 3.4 Adalimumab treatment

All 8 children were treated with adalimumab according to weight-based dosing protocols.

### 3.5 Efficacy and safety analysis

#### 3.5.1 Symptom improvement

After treatment with adalimumab, complete resolution of clinical symptoms was observed in all patients. The complete healing of all patients with oral and genital ulcers was observed. We also observed complete healing of the skin in all the patients.

#### 3.5.2 Inflammatory markers

Significant reductions in inflammatory marker levels were observed posttreatment. The erythrocyte sedimentation rate (ESR) decreased from 44.63 ± 43.48 mm/h to 10.50 ± 7.65 mm/h (*p* = 0.046), and high sensitivity C-reactive protein (hs-CRP) levels decreased from 43.87 ± 39.10 mg/L to 0.96 ± 0.67 mg/L (*p* = 0.017) (Table 2).

**Table 2:** ESR and hs-CRP in children with Behcet's disease before and after adalimumab treatment ($\bar{x}\pm s$).

Parameter	Preadalimumab	Postadalimumab	*t*	*p*
ESR (mm/h)	44.63 ± 43.48	10.50 ± 7.65	2.186	0.046
hs-CRP (mg/L)	43.87 ± 39.10	0.96 ± 0.67	3.104	0.017

#### 3.5.3 Endoscopic findings

**Figure 1:**
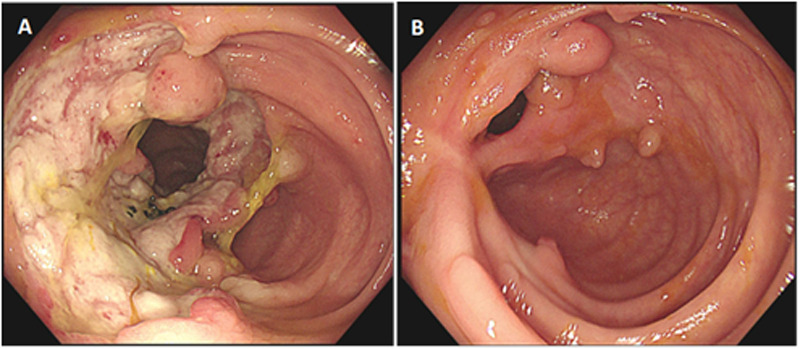
Comparison of colonoscopy findings in a pediatric patient (patient 1) with intestinal behçet's disease before and after adalimumab treatment. **(A)** Before treatment: a large circumferential ulcer with a clear boundary and covered with yellow–white exudate was observed in the ileocecal region. **(B)** After 4 months of treatment with adalimumab: scarring with white fibrous tissue was noted at the site of the previous ulcer in the ileocecal region, accompanied by multiple polypoid hyperplasia of the mucosa.

Follow-up gastrointestinal endoscopy revealed that 6 patients (patient 1, patient 2, patient 3, patient 4, patient 6, and patient 8) had completely healed ulcers (Figure 1), whereas the remaining 2 patients (patient 5 and patient 7) had ulcers reduced to 50% of their original size.

#### 3.5.4 Efficacy assessment

All 8 children received continuous treatment with adalimumab. On the basis of clinical symptoms and endoscopic findings, 6 patients (75%) achieved complete remission. The 6 patients (patient 1, patient 2, patient 3, patient 4, patient 6, and patient 8) required adalimumab treatment for a duration of 8.83 ± 3.82 months (ranging from 4 to 12 months) to achieve complete remission. After complete remission, these 6 children continued to receive adalimumab treatment.

On the basis of the clinical symptoms and endoscopic findings, 2 patients (patients 5 and 7) showed improvement. Patient 5 improved after 6 months of treatment with adalimumab, with the ulcers shrinking to 50% of their original size and no clinical symptoms. Patient 7 improved after 13 months of treatment with adalimumab, with the ulcers shrinking to 50% of their original size and no clinical symptoms. Patients 5 and 7 continued to receive adalimumab treatment.

#### 3.5.5 Adverse events

Adalimumab was well tolerated, with the duration of treatment for children ranging from 16 months to 33 months, and only 1 patient (12.5%) developed recurrent eczema on the neck. No serious adverse events, such as infections or malignancies, were reported. Routine blood tests and liver/kidney function tests revealed no abnormalities in any of the children.

## 4 Discussion

This study included 8 children with intestinal Behçet's disease, some of whom had previously received corticosteroids or immunosuppressants. Endoscopy revealed active ulcers, and some children experienced complications such as obstruction or perforation. After treatment with adalimumab, the patients' clinical symptoms improved, their inflammatory marker levels decreased, and their ulcers gradually healed. Except for one case of recurrent eczema, no other adverse events were observed during follow-up. This study provides clinical evidence for the efficacy and safety of adalimumab in treating intestinal Behçet's disease in children.

Intestinal Behçet's disease can involve multiple systems and has diverse clinical manifestations. Diagnosis requires endoscopic and imaging studies, and it is essential to exclude other causes of ulcers, such as tuberculosis, inflammatory bowel disease, and NSAID use (13). The treatment of intestinal Behçet's disease is complex and individualized, depending on the affected organs, severity, and duration of the disease. The goal of treatment is to prevent and reduce systemic inflammation. To date, corticosteroids and immunosuppressants have been the main empirical treatments for intestinal Behçet's disease; however, some patients respond poorly to these treatments.

With advancements in understanding the pathogenesis of intestinal Behçet's disease, targeted therapies have become a focus. TNF-α is a key inflammatory mediator in intestinal Behçet's disease, and TNF-α inhibitors have shown efficacy in treating this disease. Studies have demonstrated that TNF-α inhibitors can rapidly improve symptoms, reduce hs-CRP levels, promote ulcer healing, and maintain long-term remission with good tolerability. The European League Against Rheumatism recommended the use of anti-TNF-α monoclonal antibodies for severe or refractory intestinal Behçet's disease (13). While infliximab has been widely studied and shown to be effective and safe in treating intestinal Behçet's disease (14, 15), data on the use of adalimumab, particularly in children, are limited (16). For children with Behcet's disease with ocular and gastrointestinal involvement, adalimumab and infliximab are superior to etanercept monoclonal antibodies (17). A real-world prospective study from South Korea revealed that adalimumab is safe and effective in adult patients with intestinal Behçet's disease, with significant improvements in clinical symptoms and disease activity indices (9). Our study also demonstrated the safety and efficacy of adalimumab in children.

The efficacy of adalimumab in this pediatric cohort aligns with the growing body of evidence supporting the role of TNF-α as a central mediator in the pathogenesis of intestinal Behçet's disease. TNF-α not only drives systemic inflammation but also contributes to the formation of deep, penetrating ulcers characteristic of intestinal Behçet's disease. The significant reduction in inflammatory markers (ESR and hs-CRP) observed in our study underscores the potent anti-inflammatory effects of adalimumab. Furthermore, complete ulcer healing in 6 out of 8 patients suggests that early intervention with TNF-α inhibitors may prevent disease progression and reduce the risk of complications such as perforation or obstruction. This is particularly relevant in pediatric patients, where the disease can have a more aggressive course than it does in adults. The ability of adalimumab to induce mucosal healing and maintain long-term remission highlights its potential as a first-line biologic therapy for pediatric intestinal Behçet's disease, particularly in patients refractory to conventional treatments such as corticosteroids and immunosuppressants.

While the efficacy of adalimumab in adult intestinal Behçet's disease has been well documented, data in pediatric populations remain scarce. Our findings demonstrate a high rate of complete remission (75%). Notably, the safety profile of adalimumab in our cohort was excellent, with only one case of mild eczema reported. This contrasts with some adult studies, where higher rates of infections or injection site reactions have been reported (9, 10), although direct comparisons are limited by differences in outcome measures and treatment durations. The favorable safety profile in children may be attributed to their generally robust immune system and relatively few comorbidities. However, long-term follow-up studies are necessary to assess the risk of rare adverse events, such as malignancies or opportunistic infections, which have been reported in adult populations (10, 18). These findings suggest that adalimumab is not only effective but also well tolerated in patients with pediatric intestinal Behçet's disease, making it a viable option for early and aggressive disease management.

Several limitations should be noted in this study. First, as a single-center retrospective analysis, it may be subject to selection bias. Second, the sample size was relatively small, and the follow-up periods and concomitant medications varied, which may affect the assessment of short-term and long-term efficacy. However, these are common issues in retrospective studies.

## 5 Conclusion

In conclusion, adalimumab is effective and safe for treating intestinal Behçet's disease, promoting ulcer healing and improving gastrointestinal symptoms. This study provides clinical evidence for the use of adalimumab in children with intestinal Behçet's disease.

Take home messages: (i) Intestinal Behçet's disease is a rare but severe manifestation of Behçet's disease, particularly in pediatric populations. (ii) Currently, there is no cure for intestinal Behçet's disease, and the goal of treatment is to control acute episodes and reduce inflammation. (iii) Adalimumab is not only effective but also well tolerated in treating pediatric intestinal Behçet's disease, making it a viable option for early and aggressive disease management.

## Data Availability

The original contributions presented in the study are included in the article/Supplementary Material, further inquiries can be directed to the corresponding authors.
